# 3D Cell Culture in Alginate Hydrogels

**DOI:** 10.3390/microarrays4020133

**Published:** 2015-03-24

**Authors:** Therese Andersen, Pia Auk-Emblem, Michael Dornish

**Affiliations:** FMC BioPolymer AS, Industriveien 33, 1337 Sandvika, Norway; E-Mails: pia.auk-emblem@fmc.com (P.A.-E.); michael.dornish@fmc.com (M.D.)

**Keywords:** alginate, hydrogel, 3D, drug development, tissue regeneration, drug discovery, AlgiMatrix^®^, NovaMatrix^®^-3D, beads, bioprinting

## Abstract

This review compiles information regarding the use of alginate, and in particular alginate hydrogels, in culturing cells in 3D. Knowledge of alginate chemical structure and functionality are shown to be important parameters in design of alginate-based matrices for cell culture. Gel elasticity as well as hydrogel stability can be impacted by the type of alginate used, its concentration, the choice of gelation technique (ionic or covalent), and divalent cation chosen as the gel inducing ion. The use of peptide-coupled alginate can control cell–matrix interactions. Gelation of alginate with concomitant immobilization of cells can take various forms. Droplets or beads have been utilized since the 1980s for immobilizing cells. Newer matrices such as macroporous scaffolds are now entering the 3D cell culture product market. Finally, delayed gelling, injectable, alginate systems show utility in the translation of *in vitro* cell culture to *in vivo* tissue engineering applications. Alginate has a history and a future in 3D cell culture. Historically, cells were encapsulated in alginate droplets cross-linked with calcium for the development of artificial organs. Now, several commercial products based on alginate are being used as 3D cell culture systems that also demonstrate the possibility of replacing or regenerating tissue.

## 1. Introduction

The world around us, including the human body, is constructed in three dimensions. Since the 1940s, cells have been cultured, often attached to glass or plastic surfaces, essentially in two dimensions. Today, there is a need for more realistic and controllable culture systems that support cell growth, organization and differentiation essentially as found in tissues and organs. Growing cells in 3D adds a variety of aspects more physiologically significant than would be possible in 2D. A few of these are: cell culture and tumor formation of malignant cells; more relevant drug development and testing; *in vitro* culture of multi-cellular tissue for later implantation.

Despite the major differences compared to the naturally occurring 3D cell environments found in tissue, most cell culture studies *in vitro* are performed using cells cultured as monolayers (2D) on hard plastic or glass surfaces because of the ease, convenience and high cell viability associated with this culture method. However, forcing cells to adapt to an artificial flat and a rigid surface can alter cell metabolism and change or reduce functionality, thereby providing results that may not be similar to expected behavior *in vivo* [1,2]. A powerful and reliable tool for evaluation of cell behavior is gene expression data. Significant changes comparing cells cultured in 2D compared to 3D can be found associated with key biological processes such as immune system activation, defense response, cell adhesion and tissue development [3,4]. There is no doubt that 3D systems are biologically more relevant and 3D cell culture is therefore expected to also provide cellular responses that will be of higher biological relevance.

The significance and potential of *in vitro* cell culture studies are great considering the need for more cost efficient development of new drugs, time efficient treatment of cancer patients, and an understanding of developmental biology and mechanisms of stem cell differentiation. One example relates to drug development where, currently, only 12% of drugs that enter clinical trials are eventually approved for use in humans [5]. Most drugs fail due to efficacy, which likely could have been revealed at an earlier time point with more reliable cell culture models. Consequently, appropriate cell models would also reduce the need for animal trials, especially for toxicity assays [6]. Reducing the number of animal trials would also be in alignment with the principles of the 3Rs [7] (Replacement, Reduction, Refinement) which are considered an ethical framework for conducting scientific experiments using animals humanely. To better predict the clinical outcome of medical treatments such as chemotherapy, the selection of drugs can be optimized based on the response from isolated cancer cells from the patient.

There are several formats and materials available that enable 3D cell culture. We will focus on the “physical” differently shaped hydrogel formats like beads, moldable gels, injectable gels and macroporous structures. However, other technologies such as hanging drop, low-binding plastic, pyramid plates, *etc.*, are also available for culturing cells in 3D. Some macroporous scaffolds such as meshes, fibrous patches or foams, enable cell seeding throughout the thickness of the matrix and cells may be spatially organized. Such systems are, however, considered semi-3D or 2.5D [1,8] as the initial cell–matrix interaction will be more similar to what is found in 2D with cells spreading on the surface of fibers or pore walls. This is especially true for polystyrene-based 3D cell culture materials.

Nearly all cells that make up tissue reside in an extracellular matrix (ECM). The ECM consists of a complex three-dimensional (3D) fibrous meshwork of collagen and elastic fibers embedded in a highly hydrated gel-like material of glycosaminoglycans, proteoglycans and glycoproteins [1]. All together they provide complex biochemical and physical signals to the cells. A wide range of biomaterials have demonstrated applicability as matrices providing a biologically more relevant environment for cells mimicking several characteristics of the ECM such as physical, mechanical and biological properties. 3D cell culture can be defined as when cells are embedded in a scaffold or matrix and signals from the scaffold and surrounding cells can be received from all directions [1,8]. Cell to cell communication can occur in three dimensions as well. This requires that cells are first suspended in a hydrogel precursor solution and next entrapped by a gel initiation reaction forming covalently or non-covalently linked molecules [9,10]. Polymer hydrogels are considered well suited for 3D cell culture as they have similarities to natural extracellular matrix. Examples of synthetic materials with the capability of forming hydrogels are polyethylene glycol (PEG), poly(hydroxyethyl methacrylate) (polyHEMA), polyvinyl alcohol (PVA) and polycaprolactone (PCL). Natural polymers (and proteins) able to form hydrogels are alginate, chitosan, hyaluronan, dextran, collagen and fibrin where alginate hyaluronan (as a product of bacterial fermentation) and dextran represent non-animal derived materials.

Despite the homogeneous nature of synthetic polymers, their use as cell-entrapping materials has to some extent been avoided due to harsh polymerization conditions [1]. However, some initiator systems for photopolymerization of, for example, PEG-diacrylates are suitable for cell based hydrogel formation considering cytotoxicity, crosslinking efficiency and crosslinking kinetics [11]. Components of animal tissue are naturally recognized by cells due to the presence of cell binding ligands [12] and have been considered as good materials for scaffolds. However, these materials are less attractive because of a reduced degree of experimental control due to batch-to-batch variations as a result of their inherent diversity in material composition. Animal-derived materials may also have limited availability, and for use in the clinic, there are potential risks of immunogenicity and pathogen transmission; hence, obtaining regulatory approval for such applications may be challenging [8]. Natural hydrogels of non‑animal origin are of great interest because of their outstanding biocompatibility and mild gelation conditions, although limited control of gelation kinetics, inherent variations in material composition, and limited control over mechanical properties have been reported [1].

Alginate hydrogels have demonstrated high applicability as a structure for cell immobilization. Different soft and elastic hydrogels with typically 98%–99% aqueous media can be formulated at physiological conditions with preservation of cell viability and function. Since alginate microbeads were used for the first time in humans as an artificial pancreas in the 1980s [13], the polymer has been used with different cell types both *in vivo* and *in vitro*. Alginate is recognized for properties and characteristics such as its ability to make hydrogels at physiological conditions, gentle dissolution of gels for cell retrieval, transparency for microscopic evaluation, gel pore network that allows diffusion of nutrients and waste materials in addition to its non-animal origin. Culture of cells in alginate beads is well known [14], and a standard guide describing cell encapsulation in alginate is available from ASTM International [15]. Well-characterized alginates with high purity should be used to prepare hydrogels with consistent mechanical properties for cell encapsulation.

In this review, we will give an introduction to physicochemical and biological properties of alginates and the interaction of alginate hydrogels with cells. In addition, we will focus on 3D cell culture techniques and present aspects of immobilization of cells in alginate beads and new alginate‑based 3D cell culture kits commercially available for use with standard cell culture well plates.

## 2. Alginate

Commercially available alginates are extracted from harvested brown seaweeds. Significant amounts can also be produced by fermentation of bacteria, but this technology is not yet commercialized and will not be presented herein. The annual production of algal alginates is estimated to be approximately 38,000 tons worldwide and the largest volumes go to the food and pharmaceutical industry [16]. Alginates are also used as biomaterials in biomedical products for human use which are already on the market or in clinical trials. Such applications include wound healing, a bone graft substitute for spine fusion, cell therapy, and augmentation of the left ventricle wall for patients with dilated cardiomyopathy [17].

### 2.1. Alginate Structure, Chemistry and Purity

**Figure 1:**
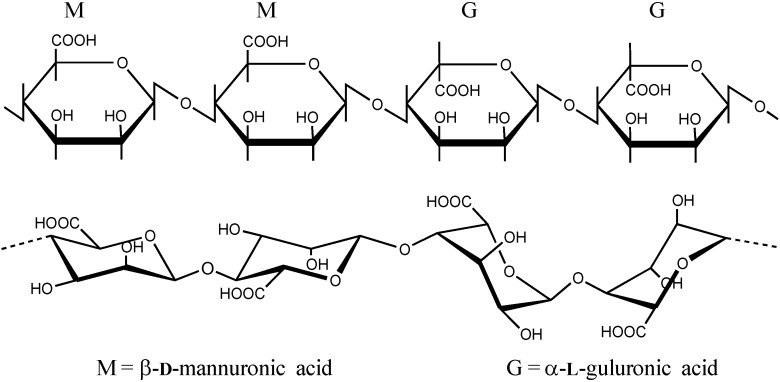
The structure of alginate shown as the segment of ..MMGG.. residues [18]. Epimerisation of the M residues changes the conformation of the sugar from ^4^C_1_ to ^1^C_4_ [19,20].

Alginates are polysaccharides which consist of linear (unbranched) 1,4 linked residues of β-**d**-mannuronic acid (M) and its C5-epimer α-**l**-guluronic acid (G) (Figure 1). The alginate molecular structure contains blocks of consecutive G or M monomers (-GGG- or -MMM-) or blocks of alternating monomers (-MGMG-). The G content of most algal alginates varies between 30% and 70%. The blocks vary considerably in length and distribution depending on from what species and part of the seaweed the alginate is extracted. The chemical composition and distribution of blocks in the alginate molecule play a major role in their capability of forming ionic gels.

At neutral pH alginate has a polyanionic character due to the pK_a_ values **d**-mannuronic and **l**‑gulronic acid of 3.38 and 3.65, respectively [21]. Hence, acidification below pK_a_ leads to insoluble alginic acid, whereas alginate molecules in solution have an extended random coil conformation due to intramolecular electrostatic repulsion between neighboring negative charges. This results in highly viscous solutions of alginate even at low concentrations where the viscosity is influenced by the ionic strength, temperature and molecular weight [21].

Commodity alginates, while having similar physicochemical properties, may contain contaminants inducing adverse cell reactions or undesired and uncontrolled cell to matrix interactions. Cells do not have receptors that recognize alginates and regular commercially available alginates can be considered as inert if they are of ultrapure quality. Impurities that should be considered and controlled in alginates for biomedical applications are presented in ASTM F 2067 and relate to the level of endotoxins, protein contaminants, elemental impurities and microbial bioburden [22]. The presence of residual endotoxins will, for example, interact with the liposolysaccharide (LPS) receptor CD14 [23]. CD14 is involved in different cell signaling pathways related to management of sepsis and can induce secretion of cytokines and upregulation of adhesion molecules. To ensure consistent cellular behavior in the presence of alginate biomaterials, the use of well-characterized and highly purified alginates is essential.

### 2.2. Alginate Hydrogels

#### 2.2.1. Ionic Gelation

**Figure 2:**
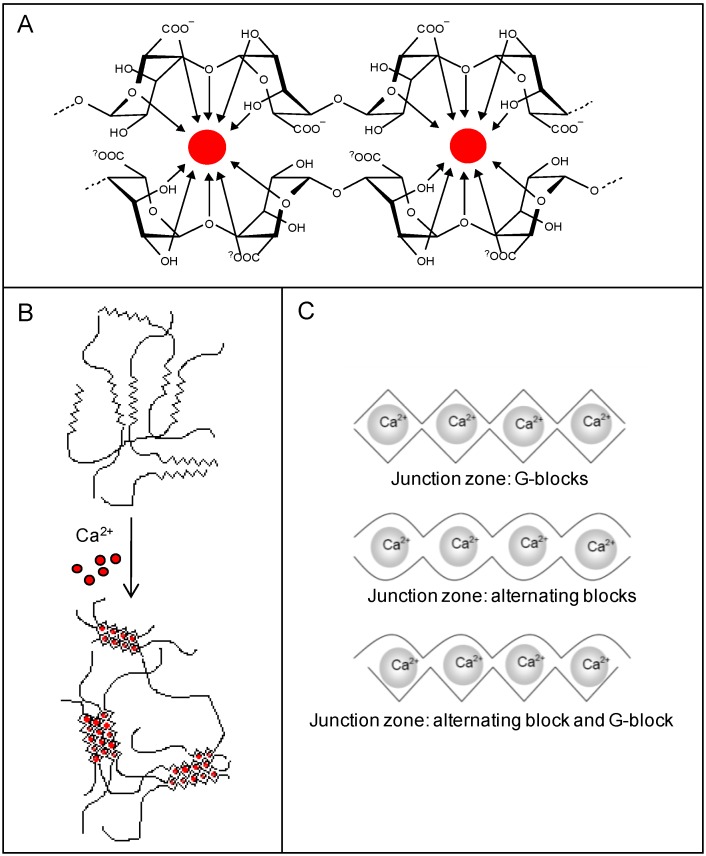
Four consecutive G-residues from two chain segments of one or two alginate molecules creating a binding site for divalent cations (**A**). Formation of an intermolecular network of alginate molecules formed in presence of gelling ions such as Ca^2+^ (**B**). Gelling ions organized in alternative junction zones (**C**) [18].

Alginates have high affinity for alkaline earth metals and ionic hydrogels can be formed in the presence of divalent cations (except Mg^2+^) [21,24,25,26,27]. Chelation of the gel-forming ion occurs between two consecutive residues (Figure 2A) and an intermolecular gel network is formed as a result of a cooperative binding of consecutive residues in different alginate chains (Figure 2B). The G-blocks are the key structural elements in alginate hydrogels, but also alternating blocks may contribute to gel formation [27]. The different junction zones in an alginate gel are presented in Figure 2C. Alginates have different affinity for divalent cations in an increasing manner as Ca^2+^<Sr^2+^<Ba^2+^ [28]. The selection of type and amount of gel forming ion will influence the resulting properties of the hydrogels and can be utilized as an important tool to optimize elasticity, swelling and stability. Additionally, gel properties can be tuned by the selection of type and concentration of alginate. In general, gel elasticity, porosity and stability increases by increasing G-content, length of G-blocks and molecular weight [27,29].

The nanoscale porosity of an alginate gel network is tunable and in the range of 5–200 nm [29]. This will enable cellular access to nutrients and removal of waste products and synthesized products such as insulin, dopamine, endostatin and nerve growth factors [30,31].

Ionically gelled alginate can be dissolved by treatment with chelating agents for divalent cations such as citrate and ethylenediaminetetraacetic acid (EDTA) or hexametaphosphate [14]. This enables gentle and fast release of cells entrapped in alginate hydrogels for further downstream processing such as flow cytometry.

Ionic cross-linking between multivalent cations and alginate takes place instantaneously. Gels are, therefore, not easily prepared by mixing these two components directly. Two main techniques have been developed to provide controlled introduction of gelling ions to alginates with a subsequent formation of a gel. The techniques are referred to as diffusion gelation and internal gelation.

Diffusion gelation is characterized by allowing gelling ions to diffuse from a large outer reservoir into an alginate solution. This is commonly used for preparation of hydrogel beads which will be formed instantaneously when an alginate solution is dripped into a solution containing gelling ions (usually CaCl_2_) [32]. This technique is described in more detail in Section 3.1.

Internal gelation is characterized by a controlled release of gelling ions from an inert source that is dissolved or suspended within the alginate solution. Examples of sources of gel forming ions include carbonate salts (SrCO_3_ or CaCO_3_), calcium EDTA, calcium citrate, calcium sulfate (CaSO_4_), calcium alginate (Ca-alginate) and calcium gluconate [27,33,34,35,36]. Release of gel forming ions can be induced by a change in pH by, for example, incorporating the slowly hydrolyzing glucono-δ-lactone (GDL), limited solubility of a calcium salt source (Ca-alginate, CaSO_4_) and/or presence of chelating agents [37]. Compared to the method of diffusion gelation, the gelling ions are here released in a controlled fashion and over a period of time from sources distributed throughout the alginate. A molar ratio between GDL and SrCO_3_/CaCO_3_ of 2:1 will provide neutral gels [38]. Examples where this technique is utilized are described in more detail in Section 3.2 and Section 3.3.

#### 2.2.2. Covalent Gelation

Covalently cross-linked hydrogels can be prepared from chemically modified alginates [39,40]. To better control the physical properties of alginate gels, covalent cross-linking has been broadly investigated [41]. A covalently cross-linked hydrogel is chemically stable and can provide different modes of stress relaxation. By covalently conjugating methacrylate groups onto the alginate backbone, covalently cross-linked hydrogels can be prepared in the presence of a photoinitiator and UV light. Cells will be evenly distributed throughout the hydrogel if the cells are suspended in the alginate solution prior to photocrosslinking [17,42]. Photocrosslinking has a number of different advantages. By comparison with ionic gelation, photocrosslinking allows formation of more stable alginate hydrogels independent of the level of gel forming ions and non-gelling ions [42]. Mechanical properties and biodegradation rates of a hydrogel can be adjusted by varying the degree of alginate methacrylation [43].

### 2.3. Alginate Derivatives

Alginates can be chemically functionalized to alter physicochemical and biological characteristics and properties. One example is mentioned in Section 2.2.2 related to preparation of covalently gelled matrices. One approach for tuning degradation of alginate hydrogels includes the use of covalently cross-linked methacrylated alginates where the linkages are hydrolytically degradable [43,44], or inclusion of linkages that can be cleavable by matrix metalloproteinases (MMP) [45]. Another approach to enhance depolymerization is by partial periodate oxidation where some of the residues along the chain are made degradable by β-elimination [46,47,48]. Other tunable properties are solubility, hydrophobicity, bioactivity *etc.* Due to the free hydroxyl and carboxyl groups distributed along the backbone, alginate is a suitable candidate for chemical modification, and these are presented in reviews by Yang *et al.* [49] and Pawar and Edgar [50]. The most important modifications of alginate hydrogels for use in combination with cells are related to the ability to tailor and control the type and degree of cell interactions. This can be achieved by covalently conjugating alginate with heparin binding peptides (HBP) or peptide sequences found in ECM proteins. Cell matrix interactions can thereby be enabled via the non-integrin receptor syndecan for HBP or integrins for ECM peptides [51,52,53,54]. ECM peptide coupled alginates will be discussed in more detail below.

#### 2.3.1. Peptide-Coupled Alginates

**Figure 3:**
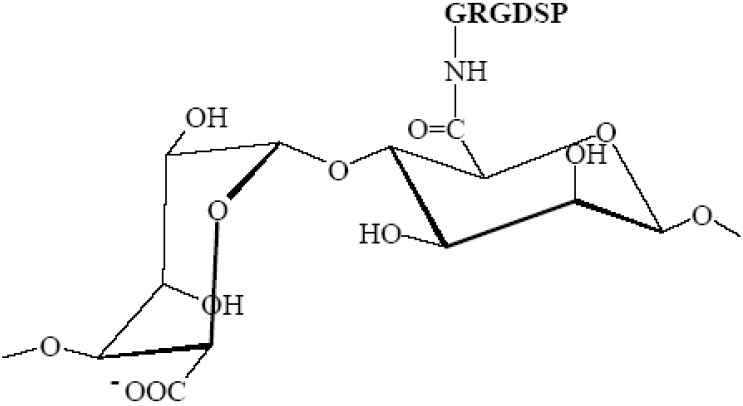
Chemical structure of RGD-alginate (arginine-glycine-aspartic acid conjugated to sodium alginate) (prepared using ISIS Draw).

The ability to modify the chemical and physical properties of alginate is a highly compelling incentive for using alginates in tissue engineering and regenerative medicine applications [55]. Cell attachment peptides, especially the sequence RGD (arginine-glycine-aspartic acid), have been shown to improve cellular adaptability to matrices, and such is also the case with alginate. Using aqueous carbodiimide chemistry, alginate can be modified by covalently grafting peptide sequences to the alginate molecule [56]. The interaction of cells with biomaterials is often mediated through cellular receptors that recognize adhesion molecules at material surfaces. One common example of such an adhesion ligand is the RGD peptide sequence, and it has been shown that RGD-coupled alginates (Figure 3) have the ability to initiate biological interactions between alginate hydrogels and cells [56,57].

**Figure 4:**
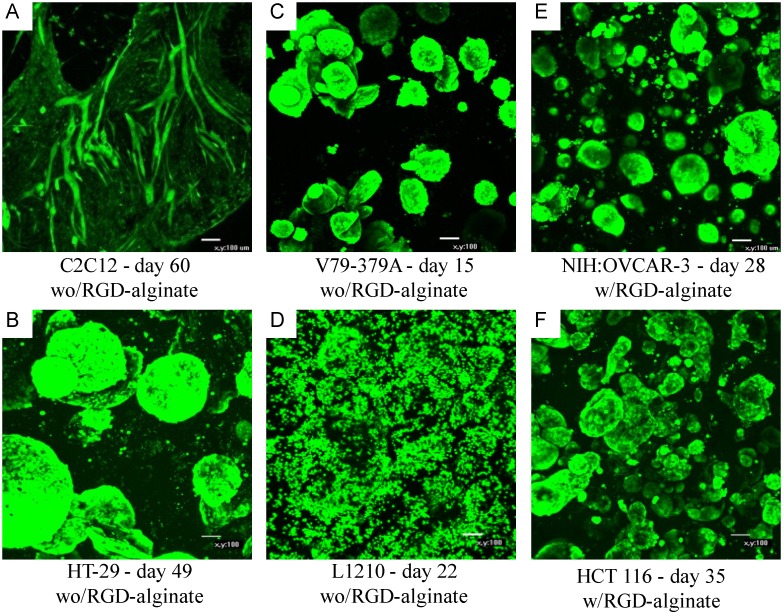
Cellular organization within the alginate gel obtained by confocal microscopy of vital stained (Calcein AM) cells. Panels A–D and E–F show cells cultured without and with RGD-alginate, respectively. C2C12: murine myoblasts, HT-29: human colorectal adenocarcinoma cells, V79-379A: Chinese hamster lung fibroblasts, L1210: murine leukemia suspension cells, NIH:OVCAR-3: human ovarian adenocarcinoma cells, HCT 116: human colorectal carcinoma cells. Magnification ×100, scale bar 100 µm. Adapted from [60].

As cells do not have receptors that recognize alginate, proliferation and differentiation of some cells within an alginate hydrogel do require signaling molecules and matrix interaction. Studies using an alginate-based scaffold for 3D cell culture (NovaMatrix^®^-3D) have shown the importance of the presence of the RGD peptide sequence for cell proliferation. Table 1 lists various established cell lines grown in the NovaMatrix^®^-3D cell culture system and if RGD was required for cell proliferation. The commercial RGD-alginate NOVATACH MVG GRGDSP was used at a concentration of 120 µM RGD obtained by dilution with non-RGD alginate. (NOVATACH MVG GRGDSP contains 0.02–0.04 µmole RGD/mg alginate, and the exact value is reported on the certificate of analysis.). For example, the non‑tumorigenic cell lines C2C12 (mouse myoblast) and MDCK (canine kidney) did not proliferate in the alginate hydrogel during the first few weeks of culture. However, if RGD was available through incorporation of RGD-alginate, then cells displayed rapid proliferation. The data [58] on cell proliferation of C2C12 myoblast cells corroborated the importance of RGD peptide on cell proliferation found by Rowley and Mooney [59]. Moreover, after several weeks of culture, the C2C12 cells started to proliferate and fuse into multinucleated myofibrils as seen in Figure 4A. Figure 4 shows confocal images of the spatial organization of different cell types that were vital stained with Calcein AM (Molecular Probes) while inside the gel [60].

**Table 1:** The importance of RGD (120 µM) as RGD-coupled alginate for cell proliferation in NovaMatrix^®^-3D (reproduced from www.novamatrix-3D.com) [58].

Cell type	Cell line ID	Source	NOVATACH MVG GRGDSP
			required for proliferation^1^
**Cell lines—Non-tumorigenic origin**			
Lung fibroblasts (Chinese hamster)	V79-379A	Former Flow Laboratories	No^2^
Myoblasts (epithelial, murine)	C2C12	ATCC CRL-1772	Yes
Fibroblasts (embryonic, murine)	NIH:3T3	ATCC CRL-1658	No^3^
Kidney epithelial cells (Madin Darby, canine)	MDCK	ATCC CCL-34	Yes
**Cell lines—Carcinoma/adenocarcinoma origin**			
Cervix (human)	NHIK 3025	Norwegian Radium Hospital	No
Ovarian (human)	NIH:OVCAR-3	ATCC HTB-161	Yes
	SKOV-3	ATCC HTB-77	No^3^
Colon (myofibroblasts, hTERT-immortalized, human)	CT5.3	Proprietary	No^3^
Colorectal (human)	LoVo	ATCC CCL-229	No^3^
	SW620	ATCC CCL-227	No
	HT-29	ATCC HTB-38	No^3^
	HCT 116	ATCC CCL-247	No
Lung (human)	A549	ATCC CCL-185	No^3^
Prostate (human)	DU145	ATCC HTB-81	No^2,3^
Breast (human)	MCF7	ATCC HTB-22	No^3^
	ZR-75-1	ATCC CRL-1500	
	MDA-MB-231	ATCC HTB-26	
	MDA-MB-361	ATCC HTB-27	
Pancreas (human)	PANC-1	ATCC CRL-1469	No^2,3^
	MIA PaCa-2	ATCC CRL-1420	No^2,3^
Leukemia (suspension, murine)	L1210	ATCC CCL-219	No^3^
	P388-D1	ATCC CCL-46	No

^1^ Cell proliferation evaluated during three weeks of culture (NOVATACH is a tradename for RGD-alginate from FMC BioPolymer). ^2^ NOVATACH MVG GRGDSP influence on cell morphology. ^3^ Cell proliferation was accelerated in presence of NOVATACH MVG GRGDSP.

Increased cell proliferation has been seen by increasing density of RGD [59,61]. Variations in nanoscale organization of RGD was shown to influence adhesion, proliferation and differentiation of preosteoblasts [61], which can be obtained by diluting RGD-alginates with different RGD densities along the chain with regular alginate. The peptide densities in alginate hydrogels can be optimized dependent on the cell culture system, but they are mainly comparable to the RGD density of commonly used biological matrices. Fischbach *et al.* [62] presented the number of RGD molecules in tumors associated ECM to be 8.6 × 10^16^ per mL matrix, which equals 143 µM. In the literature, the reported densities of peptides are presented in different ways such as µg peptide/mg polymer, µmole peptide/mg polymer, fmole/cm^2^ (2D), degree of substitution based on monomer or molecule (chain).

The presentation of the RGD peptide sequence has been shown to be an important cue regulating cellular behavior. This was demonstrated by, for example, Hsiong *et al.* [63] who compared the behavior of MC3T3-E1 preosteoblasts, human bone marrow stromal cells (hBMSCs) and D1 stem cells encapsulated in alginate hydrogels containing either linear G_4_RGDSP or cyclic G_4_CRGDSPC. The results showed that linear RGD densities promoted osteogenic differentiation of committed cells (MC3T3-E1 preosteoblasts), but not for the hBMSCs and the stem cells. However, osteoprogenitor differentiation of all cells was seen in the gels presenting the higher-affinity cyclic form of the adhesion ligand. As presented in a review of Perlin *et al.* [64], the affinity and selectivity for different types of integrin receptors varies among cell types and is dependent on the flanking amino acids of RGD, the conformation and the length of the peptide sequence. Some integrins are cell type specific, whereas others mediate different cellular behaviors such as adhesion and migration. Hence, the type of optimal RGD containing peptide sequence and RGD density will vary depending on the study, which must be seen in combination with the physical properties of the peptide-containing alginate. Other extracellular matrix peptide sequences in addition to RGD have been found to be of interest to couple to alginate such as REDV (found in fibronectin), YIGSR (found in laminin) and VAPG (found in elastin) [51,65].

## 3. 3D Cell Culture

There are several approaches and adaptations of 3D cell culture for cell immobilization. One system will likely not fit all types of experiments or cell types, but as can be seen in this section, alginate provides a great toolbox for design and optimization. There is one major difference to be aware of when comparing different techniques for formation and evaluation of multicellular spheroids. This relates to whether the spheroids that are formed are monoclonal or polyclonal in origin. Spheroids of monoclonal origin are formed from a single proliferating cell as will be the situation for cells in most hydrogel systems. Spheroids of polyclonal origin arise from an aggregating cell population and will be the situation when using low attachment plates or hanging drop techniques.

Technologies for formation of scaffolds as an alternative to cell culture on 2D plastics include beads, fibers, membranes, meshes, foams or hydrogels of different shapes and sizes [1,66]. Even 2D cell culture onto different types of scaffolds show benefits over monolayers on plastics, and valuable information about cellular responses to matrix stiffness and incorporated cell signaling factors has been obtained from such experiments [59,67,68,69]. Four technologies of alginate hydrogels are presented in this section and include beads, delayed gelation systems, macroporous scaffolds and 3D printed scaffolds. Other technologies providing 3D cell culture in alginate scaffolds are honeycomb alginate scaffolds with an aligned pore structure for improved vascularization [70], scaffolds impregnated with magnetically responsive nanoparticles for stimulation of cells and induced organization of endothelial cells into capillary-like structures [71], and alginate scaffolds with controlled nucleation of hydroxyapatite generating a composite scaffold promising for bone tissue engineering [72,73].

**Figure 5:**
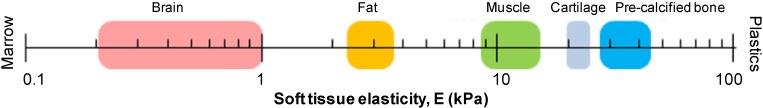
Elasticity scale of soft tissues. Adapted from reference [74]. Reprinted with permission from AAAS.

Alginate scaffolds for 3D cell culture can be tuned and optimized for a wide range of applications and cell types. In addition to tuning the interaction of cells to their surrounding hydrogel via peptide‑coupled alginates as described in Section 2.3.1, the elasticity of the gels can be controlled. By optimizing alginate concentration, type of alginate and selection of cross-linking technology (ionic or covalent) in addition to cross-linking density, alginate hydrogels can be made to match the elasticity of most types of tissue (Figure 5) and can be used to control stem cell differentiation [69,74,75,76]. The elasticity of alginate hydrogels can be made to match the elasticity of all types of soft tissue [28,74,77]. Huebsch *et al*. reported that bond formation between mammalian mesenchymal stem cells (mMSCs) and RGD peptide was regulated by both the density of available peptides (7.5–150 µM RGD concentration) and matrix rigidity (2.5–110 kPa) thereby demonstrating the importance of these two parameters [69].

### 3.1. Beads

**Figure 6:**
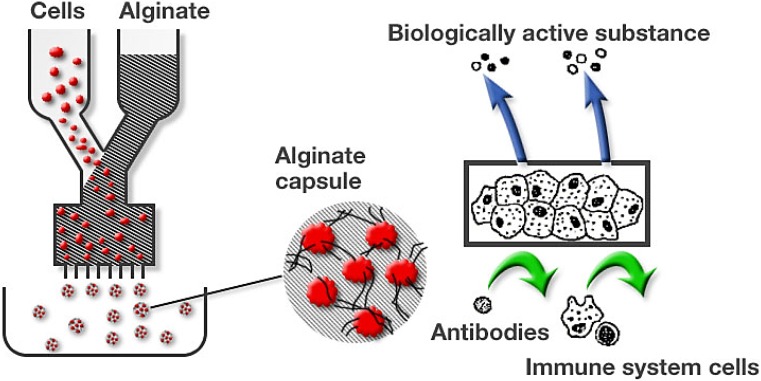
Encapsulation of cells with alginate. (From FMC internal archive.)

The technique to immobilize cells, particularly pancreatic islet cells, in calcium alginate matrices was developed by Lim and Sun at the end of the 1970s [13]. By coating the alginate gel bead with polycations like poly-L-lysine, poly-L-ornithine, or chitosan, the strength of the surface coating as well as the capsule porosity can be controlled (Figure 6) [78].

One important characteristic of alginates is their very limited inherent cell adhesion and cellular interaction. This is an advantage for cell encapsulation applications, but can be a disadvantage for tissue engineering applications. However, alginate can be modified by the addition of cell attachment peptides or other biologically active molecules (see Section 2.3.1).

Encapsulation in an alginate hydrogel has been shown to be a rapid, non-toxic, and versatile method for immobilization of macromolecules and cells. The creation of artificial organs by encapsulating cells or tissue is under study for treatment of a variety of diseases such as Parkinson’s disease, liver failure, hypocalcemia as well as chronic pain and, perhaps the most well-known example, an artificial pancreas for the treatment of diabetes (encapsulated pancreatic islets).

Most methods for encapsulation of cells or tissue in alginate gels basically involve two main steps. The first step is the formation of an internal phase where the alginate solution containing biological materials is dispersed into small droplets. In the second step, droplets are solidified by gelling, or by forming a membrane at the droplet surface.

Bead size is one of the most important parameters of alginate gel beads and capsules in biomedical applications. The appropriate size will often be a compromise. The bead itself must be large enough to contain the biological material. Larger beads are also easier to handle during washing or other treatments. In many applications involving cells, the cells should be homogeneously distributed within the internal capsular matrix. When generating beads the desired mean size and acceptable size distribution should be accounted for. The size of the beads is mainly controlled by regulating the formation of the droplet.

**Figure 7:**
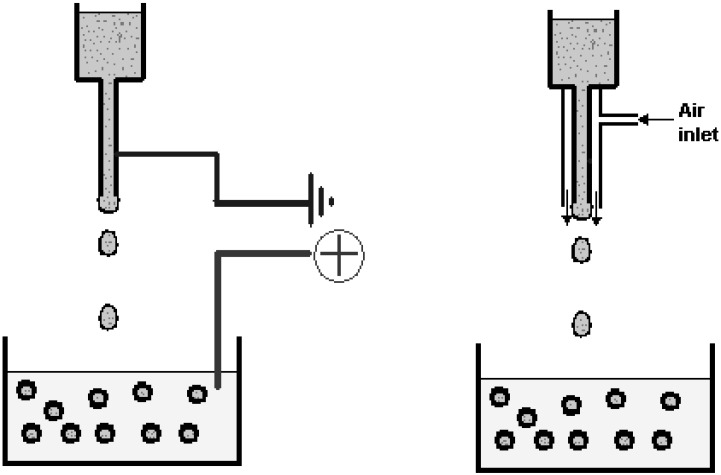
Principle of electrostatic (left) and coaxial air flow (right) bead generators [80].

Droplet size is dependent upon several factors: the size of the material to be immobilized or encapsulated (*i.e.*, single cells or cell aggregates such as pancreatic islets), the technique used to generate droplets (*i.e.*, pipette or syringe, coaxial air flow, electrostatic generator, jet-cutter, *etc.*), the viscosity of the alginate solution, and the rate of alginate flow. Generally, for biomedical applications, droplet size is regulated to give a gelled bead having a diameter of <200–1000 µm. Per unit volume, smaller beads yield a larger surface area to transplant volume, a ratio that results in enhanced survival of tissue due to better nutritional and oxygen supply. Various techniques can be used to form droplets, as described in more detail by Dulieu *et al.* [79]. These include:

• Extrusion through a needle: Beads can be made by dripping an alginate solution from a syringe with appropriate diameter needle directly into a gelling bath. While this method does not require any instrumentation, the size and size distribution of the produced beads are difficult to control.
• Coaxial air or liquid flow: The coaxial air jet system is a simple way of generating small beads (down to around 400 µm), although the size distribution will normally be larger as compared to an electrostatic system. In this system, a coaxial air stream is used to pull droplets from a needle tip into a gelling bath (Figure 7).
• Electrostatic potential: An electrostatic potential can be used to pull droplets from a needle tip into a gelling bath. The primary effect on droplet formation by the electrostatic potential is to direct charged molecules to the surface of the droplet to counteract surface tension. Using this type of instrument, beads below 200 µm and with a small size distribution may be generated. The desired bead size is obtained simply by adjusting the voltage (electrostatic potential) of the instrument. The principle for making smaller beads by electrostatic potential bead generators is shown in Figure 7.
• Vibrating capillary jet breakage: A vibrating nozzle generates drops from a pressurized vessel.
• Rotating capillary jet breakage: Bead generation is achieved by cutting a solid jet of fluid coming out of a nozzle by means of a rotating cutting device. The fluid is cut into cylindrical segments that then form beads due to surface tension while falling into a gelling bath.

Encapsulation of living cells, a form of cell immobilization, is one application for producing artificial organs and cell therapy constructs. Here, cells are mixed with alginate (osmolality is adjusted) and then the alginate + cell solution is dripped (or extruded) into a bath of calcium chloride (Figure 6). Since the ionic cross-linking reaction is instantaneous, living cells are entrapped inside an alginate hydrogel bead. The porosity of the alginate bead is such that oxygen and nutrients can diffuse into the gel while cell products, such as proteins, can diffuse out of the gel. The hydrogel is, however, porous barrier to antibodies and immune cells such as macrophages. Furthermore, it is possible to implant his alginate hydrogel ”biofactory” into an animal or man where the implant can act as a continuous production system for, for example, insulin. Implantation studies into animals and diabetic patients [81,82,83] have shown that long-term functionality.

**Figure 8:**
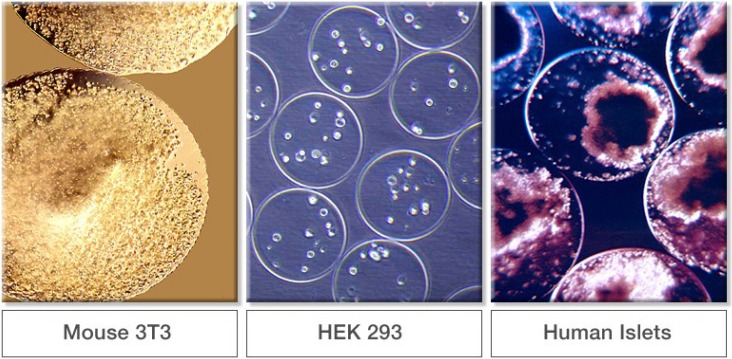
Examples of encapsulated cells in alginate. (From FMC internal archive.)

Some examples of encapsulated cells are shown in Figure 8. These photomicrographs are not of the same magnification. The panel on the left shows encapsulated genetically engineered murine 3T3 cells. Encapsulated Human Embryonal Kidney (HEK) 293 cells are shown in the center panel. Pancreatic islets encapsulated in alginate gel beads are shown in the right panel. Cells producing the anti‑angiogenic protein endostatin have been encapsulated in ultrapure alginate and implanted into the brains of dogs being treated for spontaneous brain cancer [84,85,86]. Many other cell types have been immobilized in alginate such as adipose-derived stem cells [87,88], mesenchymal stem cells [89,90], and chondrocytes [91,92].

Clinical cell therapy applications of alginate immobilized cells such as the treatment of Parkinson’s disease as well as diabetes are available at the web sites of Living Cell Technologies [93,94].

### 3.2. Delayed Gelation Systems

Delayed gelation systems where gels are formed inside the body (*in situ*) allow implantation with less invasive surgery which is easier to deliver since they will exactly fill tissue voids and defects. The mixture of gel forming ions suspended or dissolved in an alginate solution is, along with internal gelation principles, the basis of delayed gelation. Examples include mixing of an alginate solution with a suspension of calcium alginate particles [95], calcium carbonate and GDL [96] or calcium sulfate [97,98].

**Figure 9:**
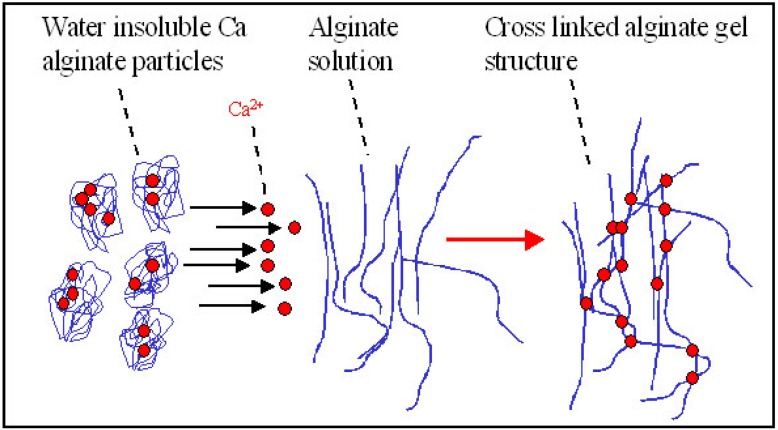
Principle of delayed gelling of a hydrogel made from alginate only [99]. By mixing a sodium alginate solution with a dispersion of insoluble calcium alginate, using for example connected syringes, a homogeneous hydrogel can be made. Upon mixing, gel forming ions rearrange between insoluble and soluble alginate molecules resulting in gel formation.

A promising approach for tissue engineering of hyaline cartilage is utilization of human mesenchymal stem cells (hMSCs). The most sufficient chondrogenic differentiation is obtained in 3D culture systems. A novel model system for 3D chondrogenic differentiation of hMSCs has recently been described [95]. Here, cells were entrapped in an alginate hydrogel formed as calcium ions diffused from calcium alginate particles subsequent to mixture with a solution of sodium alginate (see principle in Figure 9). This technology, also known as “self-gelling” alginate, allows homogeneous distribution of the cells within a hydrogel of defined size and shape. The study demonstrated how hMSCs were differentiated resulting in up-regulation of a large number of genes associated with hyaline chondrogenesis which, for example, may be used to repair potential lesions of hyaline cartilage [95].

### 3.3. Macroporous Scaffolds

Macroporous scaffolds may allow larger constructs as the mass transfer of nutrients, oxygen and waste removal will be enhanced [100]. The structure does also have predefined dimensions and they can better withstand deformation compared to compact hydrogels [101]. Also, the pore size and porosity can be tuned and optimized for the specific application and cell type [102,103,104]. However, a major challenge with such scaffolds may be cell seeding efficiency and cell distribution, as the pores are often either too small to let cells in or too large to retain cells inside. A variety of approaches have been investigated to overcome this challenge utilizing, for example, cell seeding devices [105], bioreactors [106], centrifugal force [107] and vacuum [108,109].

**Figure 10:**
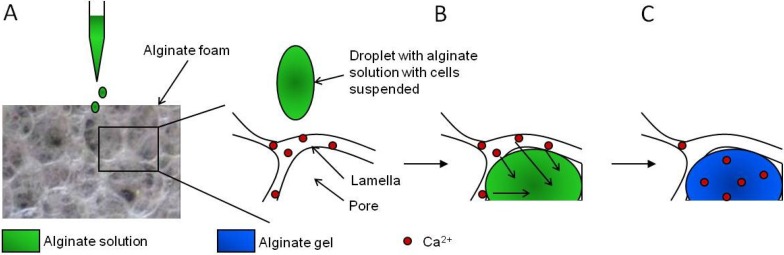
Schematic presentation of the steps for *in situ* gelation in macroporous alginate scaffolds. (**A**) Am alginate solution with cells is applied on top of a dry scaffold containing calcium ions, (**B**) rehydration of the scaffold by the alginate solution filling its pores and diffusion of calcium ions from the foam lamellas to the absorbed alginate, and (**C**) formation of a calcium cross-linked alginate hydrogel inside the pores of the foam. From reference [77].

Two alginate based macroporous systems for cell culture are available, AlgiMatrix^®^ and NovaMatrix^®^-3D from Thermo Fisher Scientific/Life Technologies (Carlsbad, CA, USA) and FMC BioPolymer/NovaMatrix (Sandvika, Norway), respectively. Both systems are based on ionically gelled and dried macroporous scaffolds and are available as sterile discs pre-filled in different formats of standard cell culture well plates [77,110]. Both scaffolds turn into hydrogels upon rehydration following cell seeding. Further, cells can be stained and microscopically evaluated while entrapped, the gels can be dissolved for easy retrieval of cells and multicellular structures and their gel elasticity is tunable. The main differences between these sponge‑like matrices are the seeding technique and ability to control cell to matrix interactions. A concentrated suspension of cells in culture media is absorbed by the dry AlgiMatrix^®^ scaffold as it is applied on the top surface and cells will be entrapped inside the porous structure. Centrifugation may be utilized to ensure a more homogeneous distribution of cells throughout the scaffold. For NovaMatrix^®^-3D, cells are applied on top of the scaffold suspended in an alginate solution dissolved in cell culture media. As shown in Figure 10, the foam structure is utilized as a scaffold and source of gel forming ions to induce gelation of the applied alginate solution. When the pores are filled with the alginate solution, a hydrogel is subsequently formed *in situ*. This technique ensures a cell seeding efficiency of about 100% and an even distribution of cells throughout the thickness of the gel. NovaMatrix^®^-3D cell culture kits are also available with RGD-coupled alginate which is added to the dry sponge together with the cells. See Section 3.2 for examples of cellular responses with or without RGD-coupled alginate.

### 3.4. Alginate as a Bioink and 3D Bioprinting

3D printing as a technology is available in industrial and home-use applications. The ability to construct customized three dimensional structures on demand using relatively simple materials is leading to a boon in manufacturing sectors. The application of 3D printing technology in the fields of tissue engineering and regenerative medicine has already begun [111]. Bioprinting uses biocompatible materials and cells to form a variety of 3D formats where cell function and viability are preserved within the printed construct. Various 3D bioprinting technologies can already form vascular-like tubes [112], artificial skin [113], cartilage [114], and a wide range of tissue constructs also including stem cells [115]. A public workshop was hosted by the U.S. Food and Drug Administration (U.S. FDA) in October of 2014 under the title “Additive manufacturing of medical devices: An interactive discussion on the technical considerations of 3D printing”. The workshop agenda, participants and presentations held at this workshop are available at the U.S. FDA web sites [116].

3D bioprinting techniques such as ink-jet and extrusion have the need for biocompatible “inks”. Alginate has shown particular relevance as a bioink due to its compatibility with cells, ease in forming cross-linked hydrogels, and the ability to control biodegradation. Khalil and Sun demonstrate bioprinting of 3D tissue constructs using alginate and endothelial cells [117] and alginate stabilized with gelatin was a suitable matrix for 3D bioprinting of bone-related SaOS-2 cells [118,119]. Common to these reports is a high (>80%) cell viability following bioprinting. These reports also show two different approaches in the design of alginate as a bioink. Khalil and Sun [117] use a multinozzle system that prints alginate + cells and overlays with calcium chloride in order to induce gelation. The addition of a low-melting gelatin together with an alginate solution forms a gel when the solution printed at 37 °C cools. Moreover, addition of a calcium poly phosphate salt or bioglass to the cell‑containing hydrogel led to enhanced biomineralization by SaOS-2 cells [118,119].

Using 3D printing technology and alginate as a bioink, Zhao *et al.* show the advantage of printing Hela cells to form an *in vitro* cervical tumor model in order to study disease pathogenesis and enable new anti-cancer drug discovery with a more relevant physiological disease model [120]. This report used gelatin together with alginate to initiate gelation prior to printing. The printed construct was further strengthened after printing by subsequent addition of a calcium salt solution. The authors included fibrinogen in the gelatin/alginate formulation to mimic ECM components. Printed HeLa cells formed spheroids which were shown to be more resistant to paclitaxel treatment than HeLa cells grown as a 2D cell culture.

By oxidizing alginate, a known technique to “build in” biodegradability [121], Jia *et al.* demonstrate the interaction of alginate viscosity and density on printability while biodegradability of printed scaffolds containing human adipose-derived stem cells was also described [122].

Optimization of alginate for use in different printing technologies is, however, necessary. For inc-jet types of printing, droplet formation is impacted by alginate molecular weight, solution viscosity, monomer composition (if ionic cross-linking is to be used to form a gel), and purity which impacts on biocompatibility. Xu *et al.* studied the characteristics of the droplet formation process using alginate viscosity and shear rate [123]. Furthermore, Gasperini *et al.* present a bioprinting techniques based on electrohydrodynamic processes to jet droplets of alginate containing cells [124].

### 3.5. Cryopreservation

Simple cell and tissue preservation techniques have disadvantages including limited shelf-life, high cost, risk of contamination or generic drift [125]. A more tangable option is cryopreservation where cells are preserved by cooling them to low temperatures typically in liquid nitrogen (−196 °C). At such low temperatures, biological activities of the cells are effectivly stopped. These includes the biochemical reactions that would lead to cell death under normal conditions and damage caused by the formation of ice crystals. Cryopreservation provides a valuable means for storing cells and tissues for future use. However, certain drawbacks exist, including damage that occurs to the cells during the freezing and/or thawing processes and the need to culture the cells after thawing to ensure that they have recovered properly. Such drawbacks limit the value of cryopreserved cells, particularly in situations where it is desirable to use the cryopreserved cells immediately or shortly after they have been thawed. There exist several methods to deal which such problems. One alternative is to suspend the cells in an alginate solution prior to cryopreservation. The cells can then be encapsulated (see Section 3.2) after thawing and used for their desired purpose without the need to culture the cryopreserved cells. Alternatively, cells are entrapped in hydrogels before they are cryopreserved [126,127]. This method is based on the discovery that cryopreserved cells that have been thawed, immediately suspended in alginate, and after encapsulation remain viable and are ready to be used [128]. To obtain off-the-shelf availability, distribution and storage of constructs, sterility testing and quality control, preservation of cells and tissues is vital [129].

## 4. Future

Developing 3D cell culture technology will lead to more physiologically relevant and likely more predictive approaches to organogenesis, tissue morphology, the importance of hypoxia, drug discovery, cell-based assays, and reduced animal use. The ability of 3D cell culture systems to mimic tissue structures, either from single cells or co-cultures, is a great advance from 2D monolayer cultures. Cell–cell communication and differentiated cellular function are more relevant in 3D and the impact of 3D cultures on predicting efficacy of drug treatments to actual *in vivo* response is great.

### 4.1. Drug Discovery

Allowing cells to acquire a more natural phenotype when grown in 3D as opposed to 2D is a great advantage. This is especially true for the field of drug discovery where countless examples have been shown of the mismatch between *in vitro* drug effect and *in vivo* drug efficacy.

#### 4.1.1. Cancer

Already in 1990 an alginate culture method was used to test the effects of vincristine and 5‑fluorouracil on HT-29 human colon carcinoma cells [130]. Creating a more clinically relevant model of tumor biology has been a prime impetus for developing 3D culture systems. Burdett *et al.* [131] described the superiority of 3D over 2D cell culture where mimicking tumor behavior and drug resistance often seen *in vivo* is important. AlgiMatrix^®^ is a commercial alginate-based product for 3D cell culture. Godugu *et al.* [110] demonstrate the possibility of using this culture system as an *in vitro* tumor model for anticancer drug screening. They treated several human non-small cell lung cancer cell lines (A549, H460, and H1650) with several anticancer drugs used in the clinic.

#### 4.1.2. Safety and Toxicology

HepG2 liver cells have been encapsulated in sterile alginate hydrogels and used to demonstrate their capability to metabolize a coumarin pro-drug in a manner similar to *in vivo* hepatic metabolic activity [132]. Another hepatic cell line, Huh-7, cultured in an alginate hydrogel showed cellular organization and hepatocyte architecture with respect to cell polarity, cell junctions and the appearance of bile canaliculi. The alginate-encapsulated Huh-7 cells also expressed specific hepatitis C virus receptors indicating that this 3D culture system may be useful in viral studied and liver tissue engineering [133]. Alginate encapsulation of hepatocytes provides protection from shear stress for hepatocyte aggregates in a 3D bioreactor cultures system [134]. In addition, the alginate hydrogel seems to provide the cells with a good support for extracellular matrix deposition.

### 4.2. Tissue Engineering and Regenerative Medicine

#### 4.2.1. Skin

Establishing normal physiology and function in a traditional 2D *in vitro* cell culture of skin is almost impossible. The advent of organotypic culture systems does allow approximation of skin complexity. 3D culture of skin allows dermatological studies which would otherwise be unsafe for animals and humans such as validating the mechanisms of skin diseases and testing the therapeutic potential of experimental drugs [135]. Developing a 3D *in vitro* human skin co-culture model has shown promise for detecting skin irritants as an alternative to *in vivo* animal testing [136].

#### 4.2.2. Cartilage

Specific signature gene cluster regulation was seen during *in vitro* chondrogenic differentiation of human bone marrow-derived mesenchymal stem cells which were immobilized in a self-gelling alginate hydrogel. Upregulation of transcription factor genes as well as a signature cluster of extracellular matrix genes occurred during chondrogenesis while gene clusters involved in immune response, blood vessel development, and cell adhesion were downregulated [95]. Marker genes identified in this study show that stem cells can be directed to produce hyaline cartilage when immobilized in 3D alginate hydrogels.

Immobilizing cells with chondrogenic potential in an alginate hydrogel has shown that neocartilage can be formed by mesenchymal stem cells [137]. Here, production of not only type II collagen but also assembled fibrils was dependent on cell seeding density. When cells were seeded at a high density, fibril assembly and procollagen processing occurred at a distance from the cell surface.

#### 4.2.3. Cardiac

Cardiac tissue engineering may involve the regeneration of myocardial tissue by first immobilizing stem cells in a scaffold or matrix *in vitro* and then placing such a scaffold on, or within, the damaged cardiac tissue. Immobilizing myocardial stem cells within a scaffold ensures that they will remain within the cardiac tissue after implantation. Ceccaldi *et al.* [138] has studied the influence of alginate composition on mesenchymal stem cells in alginate scaffolds. Their conclusion was the G-rich alginate hydrogels provided the most appropriate milieu for MSCs intended for cardiac therapy. Levit *et al.* [139] have shown similar results where alginate-encapsulated human mesenchymal stem cells were placed onto a rat heart as a hydrogel patch. The alginate hydrogel retained the MSCs and led to an improvement of cardiac function following induced myocardial infarct.

## 5. Conclusions

Culturing cells in three dimensions will soon be the preferred way to investigate cell–cell interactions, growth into tissue, mechanisms of stem cell differentiation, and improved drug efficacy, to name a few areas. Various materials are available to enable 3D cell culture among which is the polysaccharide alginate. Immobilizing cells in alginate hydrogels is a mild process that occurs under physiological conditions. In addition, cells can be retrieved from alginate hydrogels by a simple de‑gelling process that does not require disaggregation of multi-cellular structures. Alginate can be modified by the attachment of peptides that mimic extracellular matrix proteins, such as RGD, thereby allowing immobilized cells to seemingly interact with the alginate hydrogel. We have shown here that some cells actually require the presence of RGD in order to proliferate and form 3D structures.

Encapsulating cells in alginate hydrogel droplets was first described in the 1980s and various formulations are still under investigation for constructing artificial organs for, for example, treatment of Type I diabetes. Recently, two commercial alginate-based 3D cell culture systems have made their appearance. Cells are immobilized in an alginate foam-like scaffold and can then proceed to grow in three dimensions. Publications describing these 3D cell culture systems have begun to appear demonstrating their utility in several areas. Especially important for the use of alginate in 3D cell culture is the ability to change the physical characteristics of the hydrogel by changing the amount or type of gelling ions and/or alginate. One can now tailor-make an environment to which cells can adapt or differentiate.

In fields as diverse as tissue engineering and drug discovery, alginate-based 3D cell culture systems show a significant advantage over classical 2D culture techniques. In addition, automation of 3D culture techniques, especially for high throughput screening will greatly increase the use of culturing cells in this manner. Although most *in vitro* cell-based assays were originally designed using 2D cell cultures, it will be important to validate assays using a 3D culture system. New or adjusted detection chemistries may need to be developed in order to optimize the 3D cell model. This should not be detrimental to the use of 3D culture systems but rather an opportunity to improve and customize assay systems for multi-cellular structures.

The future promises ingenuity in adapting 3D culture systems into the fields of regenerative medicine. Supporting and improving cardiac function after infarct, correcting osteoarthritic cartilage degradation, and providing artificial skin for *in vitro* safety studies are among the fields 3D culture can bring new products and ideas. Finally, the adaptation of 3D bioprinting using an alginate-based bio-ink shows great promise. Patient-specific printed constructs can soon be made using alginate and a patient’s own cells. The availability of a commercial ultrapure, low endotoxin sodium alginate as well as peptide‑coupled alginate allows discrete cell signaling to be applied during 3D cell growth.
